# Crystallization of nanoparticles induced by precipitation of trace polymeric additives

**DOI:** 10.1038/s41467-021-22950-2

**Published:** 2021-05-13

**Authors:** Yiwen Qian, Alessandra da Silva, Emmy Yu, Christopher L. Anderson, Yi Liu, Wolfgang Theis, Peter Ercius, Ting Xu

**Affiliations:** 1 Department of Materials Science and Engineering, University of California, Berkeley, CA USA; 2 Materials Sciences Division, Lawrence Berkeley National Laboratory, Berkeley, CA USA; 3 Nanoscale Physics Research Laboratory, School of Physics and Astronomy, University of Birmingham, Birmingham, UK; 4 Department of Chemistry, University of California, Berkeley, CA USA; 5 Molecular Foundry, Lawrence Berkeley National Lab, Berkeley, CA USA

**Keywords:** Nanoparticles, Synthesis and processing

## Abstract

Orthogonal to guided growth of nanoparticle (NP) crystals using DNA or supramolecules, a trace amount of polymeric impurities (<0.1 wt.%) leads to reproducible, rapid growth of 3D NP crystals in solution and on patterned substrates with high yield. When polymers preferentially precipitate on the NP surfaces, small NP clusters form and serve as nuclei for NP crystal growth in dilute solutions. This precipitation-induced NP crystallization process is applicable for a range of polymers, and the resultant 3-D NP crystals are tunable by varying polymeric additives loading, solvent evaporation rate, and NP size. The present study elucidates how to balance cohesive energy density and NP diffusivity to simultaneously favor nuclei formation energetically and kinetic growth in dilute solutions to rapidly crystalize NPs over multiple length scales. Furthermore, the amount of impurities needed to grow NP crystals (<0.1%) reminds us the importance of fine details to interpret experimental observations in nanoscience.

## Abstract (web summary)

Growing nanoparticle crystals typically requires strict control over interparticle interactions and assembly. Here, the authors show that a trace amount of polymeric impurities induces reproducible, rapid growth of high quality 3D nanoparticle crystals in solution and on patterned substrates.

## Introduction

Crystallization is a ubiquitous process seen in most, if not all, classes of matter. Nanoparticles (NPs) are ideal systems for visualizing and understanding crystallization processes^1,2^ and can serve as building blocks for new classes of materials^3,4^. A dazzling array of NP crystals has been achieved by engineering complimentary interactions, e.g., by attaching DNA ligands^5–7^. To achieve highly ordered NP crystals, adequate system mobility must be maintained during the assembly process. While the initial stages of the nucleation process are still open to debate, it is commonly accepted that the critical nucleus size depends on the balance between the NP/solvent interfacial interactions and the NP/NP cohesive energy stored within the nucleus^8^. Nuclei are energetically stable at elevated NP concentrations^9^ and/or with strong ligand interactions. The NP mobility scales inversely with the NP concentration and the strength of the ligand pair interactions. Different strategies have been employed to expand the processing window for NP crystallization, including controlled solvent evaporation, interface-mediated assembly^10–12^, emulsion-based assembly^13,14^, and slow cooling of concentrated/supersaturated solutions^15^. However, it remains challenging to control the crystallization kinetics so that high-quality NP crystals can be rapidly and reliably fabricated.

Precipitating agents such as salts, solvents, and polymers have been used to drive and accelerate the crystallization of small molecules and proteins^16–19^. These additives can alter the molecular interactions to either stabilize the intermediate phase^19,20^ or to reduce the solubility of the crystallizing species^21^. Adapting this concept for NP crystallization may lead to control over the assembly kinetics and pathways, but has not yet been experimentally explored.

**Fig. 1:**
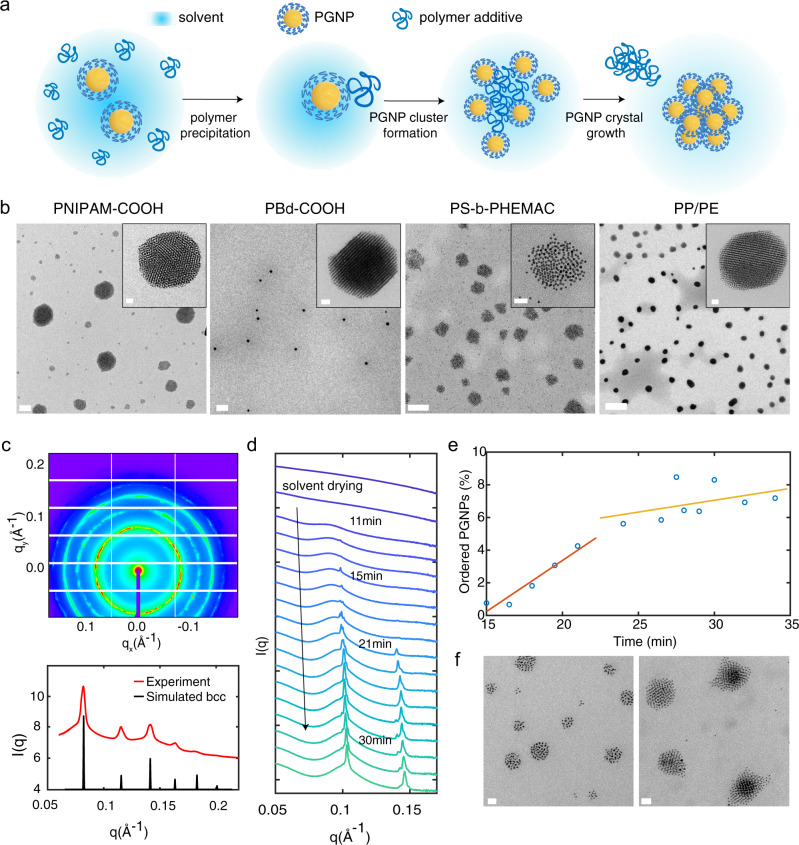
Polymer precipitation-induced PGNP crystallization in solution during solvent evaporation. **a** Schematic of polymer precipitant-induced PGNP crystallization: the added polymers precipitate onto PGNP surfaces to form small PGNP clusters that grow into crystals. **b** Representative TEM images of PGNP assemblies of (1.7k) Au-PS_0.70_ NPs with various polymer precipitants. See “Methods” for detailed polymer information. Scale bars are 100 nm for PNIPAM-COOH and PS-b-PHEMAC, 1 μm for PBd-COOH and PP/PE, and 20 nm for all inset images. **c** SAXS results of PGNP crystals in a toluene solution of (5.3k) Au-PS_0.60_ NPs with PP/PE. A simulated scattering profile of a perfect bcc lattice is shown for reference. **d** In situ SAXS studies of toluene solutions of (1.7k) Au-PS_0.70_ NPs and PP/PE during the drying process. Plots are offset for clarity. **e** PGNP crystal growth in a toluene solution of (1.7k) Au-PS_0.70_ NPs and PP/PE over time. Data points reflect the peak integration ratio between the sharp diffraction peak at q ~ 0.1 Å^−1^ in **d** from PGNPs in crystals and the broad peak from PGNPs in the solution. **f** TEM images of (1.7k) Au-PS_0.70_ NP assemblies with PP/PE upon fast solvent removal. Scale bars are 20 nm. PGNPs were dispersed at ${\varnothing }_{{\mathrm{NP}}}=0.1{\rm{vol}}. \%$ with ${\varnothing}_{{\mathrm{PP}}/{{\mathrm{PE}}}}$ = $0.08{\rm{vol}}. \%$ and all other polymers at ${{{\varnothing }}}_{{\mathrm{polymer}}}$ = $0.2{\rm{vol}}. \%$.

Here, we hypothesize that by introducing polymers with poor solubility, NP surfaces may be the preferred precipitating sites and the adsorbed polymers gradually change the NP surface chemistry. To minimize nonfavorable NP-solvent interactions, NPs form small clusters, which subsequently act as nuclei to initiate NP crystallization within a dilute solution (Fig. 1a). Given sufficient NP mobility, rapid NP crystallization is realized, and the more attractive ligand–ligand interactions lead to desorption of the polymer precipitates to achieve a high degree of crystalline order. Here, we test this hypothesis and experimentally realize polymer precipitation-induced crystallization of polymer-grafted NPs (PGNPs). 3D PGNP clusters can be obtained rapidly (in a few to tens of minutes) using a range of polymers, including impurities from plastic containers. The polymeric precipitants indeed lead to small PGNP clusters with poor local order. These clusters subsequently grow into large 3D PGNP crystals. Detailed tomography analysis of a 3D PGNP crystal with single-particle resolution confirms the presence of defects and reduced positional order of NPs at the surfaces and edges, suggesting rather weak cohesive energy and slow local NP diffusion. This precipitating additive approach is facile where the PGNP crystal formation depends on polymeric additive loading, solvent evaporation rate, PGNP size, and nucleation sites. As an example, a patterned substrate is used to grow hierarchically ordered arrays of PGNP crystals with controlled size, location, and orientation.

## Results

### Polymer precipitation leads to PGNP crystallization in solution

PGNPs were chosen as building blocks due to their programmability and diverse ligand chemistries^22–29^. Gold NPs grafted with thiol end-functionalized polystyrene (PS) ligands were well dispersed in toluene (Supplementary Figs. S1–5 and Supplementary Table 1). A series of polymers were employed as precipitates with nonfavorable interactions with toluene and the PS ligands. Figure 1b shows the transmission electron microscopy (TEM) images of PGNP crystals in the presence of carboxylic acid terminated poly(N-isopropylacrylamide) (PNIPAM-COOH, 6.3 KDa), carboxylic acid terminated polybutadiene (PBd-COOH, 2.5 KDa), and PS-b-poly(cholesteryloxycarbonyloxy ethyl methacrylate) (PS-b-PHEMAC, 29 KDa-b-28 KDa), respectively. Control experiments without polymer precipitants confirmed the absence of 3D NP crystals; only 2D superlattices were produced (Supplementary Fig. 6), consistent with previous results^24^. Interestingly, we found that polymers dissolved from a polyolefin centrifuge tube were the best precipitants, leading to rapid and reliable growth of high-quality 3D PGNP crystals. The polymer additive, hereafter called PP/PE, was chemically identified as a low molecular weight, noncrystalline polypropylene (PP) with ~10% of polyethylene (PE) as detailed in Supplementary Figs. S7–9. For homopolymers to act as precipitants, their molecular weight needs to be fairly small, typically less than ~8 KDa. See detailed information in Supplementary Table 2. Considering the polymer chain conformation in solution during precipitation and inter-NP distance at dilute concentrations, the bridging mechanism where a polymer chain acts as a linker to bring NPs together is highly unlikely. Rather, the results are consistent with the hypothesized mechanism shown schematically in Fig. 1a, where the adsorption footprint of the polymeric precipitant remains small enough to gradually modulate the NP-solvent interactions.

Au PGNPs (diameter = 3.8 ± 0.4 nm), denoted by (5.3k) Au-PS_0.60_ NPs (with PS number-average molecular weight Mn = 5300 g mol^−1^, grafting density ∑ = 0.60 chains nm^−2^), were dispersed in toluene at ${\varnothing }_{{\mathrm{NP}}}=0.1{\rm{vol}}. \%$. PP/PE was added at ${\varnothing }_{{\mathrm{PP}}/{\mathrm{PE}}}$ = $0.08{\rm{vol}}. \%$. As the toluene evaporated, small-angle X-ray scattering (SAXS) profiles showed multiple well-defined diffraction peaks with peak positions at ${q}_{n} /{q}_{1}$ = 1: $\sqrt{2}$: $\sqrt{3}$: 2 : $\sqrt{5}$: $\sqrt{7}$, consistent with a body-centered-cubic (bcc) structure (Fig. 1c). In situ SAXS was carried out to monitor the PGNP assembly during solvent evaporation (Fig. 1d and Supplementary Fig. 10). The solution contained Au PGNPs, denoted by (1.7k) Au-PS_0.70_ NPs (with PS Mn = 1680 g mol^−1^ and ∑ = 0.70 chains nm^−2^) and PP/PE at volume fraction ${\varnothing }_{{\mathrm{PP}}/{\mathrm{PE}}}=0.08 \%$. At ${\varnothing }_{{\mathrm{NP}}} \sim 23 \%$ (*t* = 11 min), the scattering profile showed a broad peak, corresponding to an average interparticle distance of 6.8 nm. This peak originated from the poorly ordered PGNP clusters since the average PGNP separation distance is ~7.8 nm at this ${\varnothing }_{{\mathrm{NP}}}$. At *t* = 15 min, an additional sharp diffraction peak appeared at ${q}_{1}=0.1{0\AA }^{-1}$. The peak intensified with a second-order peak appearing at ${q}_{2} / {q}_{1}=\sqrt{2}$, confirming the formation of well-ordered PGNP assemblies. The intensity of the diffraction peaks increased and shifted to higher *q* positions as the solvent evaporated. The final interparticle distance was 6.1 nm and the estimated domain size, derived from the peak width, was ~450 nm. Thus, the in situ solution SAXS studies show a reduction in the average inter-NP distance from 7.8 nm in solution to 6.8 nm in poorly ordered NP clusters, and 6.1 nm in NP crystals. The broad and the sharp diffraction peaks were deconvoluted to probe the PGNP crystal growth, as detailed in “Methods.” As the solvent evaporated, an increasing fraction of PGNPs assembled into ordered structures. NP crystals grew rapidly first and then growth slowed down as shown in Fig. 1e. This can be attributed to the depletion of PGNPs and the decrease in the PGNP diffusivity as the solvent evaporated and again highlights the importance of crystal growth within dilute solutions.

By using a small amount of solution (<5 μL), the self-assembly process was quenched at an early stage. Both NP assemblies with high crystalline order and poor order were seen using TEM. Image analysis confirmed that NP assemblies less than 30 nm in diameter consistently showed poor structural ordering (Fig. 1f), while larger NP clusters exhibited crystalline structures. Thus, the poorly ordered NP clusters likely still contain polyolefin precipitants, but nevertheless can act as nuclei for subsequent NP crystallization. Recent work showed that an amorphous, liquid-like intermediate phase exists within the crystallization process, in agreement with our experimental observation^2^. Further SAXS and TEM analysis confirmed that the inter-NP distance remained the same between the final PGNP assemblies with and without added polyolefins. In conjunction with the fact that the ligand–ligand interaction is much more favorable than the ligand-polymer interaction, it is reasonable to conclude that polyolefins were excluded during subsequent crystallization process.

### Effects of polymer precipitates and solvent evaporation on crystal growth

**Fig. 2:**
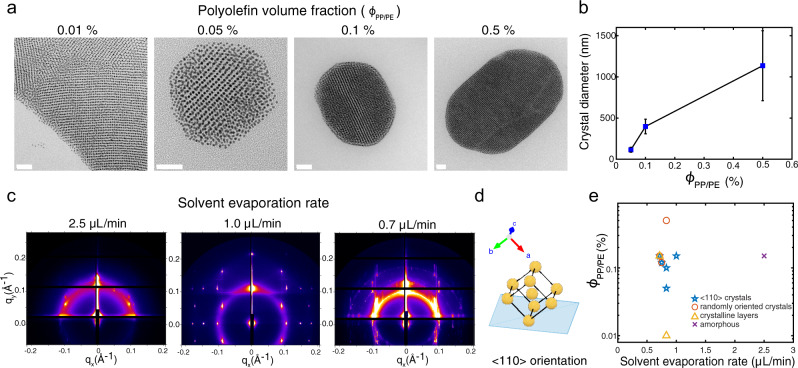
Effects of polyolefin loading and solvent evaporation rate on crystal growth. **a** TEM images of the self-assembled (5.3k) Au-PS_0.60_ NPs with different volume fractions of PP/PE. Scale bars are 50 nm for ${\varnothing }_{{\mathrm{PP}}/{\mathrm{PE}}} \sim 0.01 \%$, $0.05 \%$, and $0.1 \%$ and 200 nm for $0.5 \%$. **b** Crystal size as a function of polyolefin loading. Crystals were assembled using (5.3k) Au-PS_0.60_ NPs with different volume fractions of PP/PE. Error bars are standard deviations computed from at least 50 crystal measurements. **c** Two-dimensional GI(T)SAXS patterns of the assembled (1.7k) Au-PS_0.70_ NPs with PP/PE at ${\varnothing }_{{\mathrm{PP}}/{\mathrm{PE}}} \sim 0.15 \%$ on Si substrates with different solvent evaporation rates. Crystalline layers, substrate-oriented crystals, randomly oriented crystals, and amorphous assemblies were all observed. Incident angle $\alpha =0.8^\circ$ for a solvent evaporation rate of 1.0 μL min^−1^ and $\alpha =0.14^\circ$ for other conditions. **d** A bcc lattice with (110) planes oriented parallel to the substrate. **e** Phase diagram of the assembled morphologies as a function of solvent evaporation rate and polyolefin concentration.

The amount of polyolefin added affects the PGNP crystal size. Figure 2a shows representative TEM images of the crystals at different PP/PE loading (${\varnothing }_{{\mathrm{PP}}/{\mathrm{PE}}}$). At low ${\varnothing }_{{\mathrm{PP}}/{\mathrm{PE}}}$ ($< 0.01 \%$), layered 2D superlattices with small grain sizes were seen. 3D PGNP superlattices formed when ${\varnothing }_{{\mathrm{PP}}/{\mathrm{PE}}}$ reached $0.05 \%$. At ${\varnothing }_{{\mathrm{PP}}/{\mathrm{PE}}}$ ~ $0.5 \%$, PGNPs assembled into larger crystals with multiple grains. As more polyolefin was added, there was an increase in the average crystal diameter and size distribution (Fig. 2b and Supplementary Fig. 11). Systems of larger assemblies have a smaller total surface area, reducing the nonfavorable interactions between the PGNP surface and polyolefins. The fact that more polyolefins led to larger crystals suggests that after the polyolefins are excluded from the bulk crystal, they interact with the crystal surface and control the final crystal size. This is consistent with the unchanged interparticle distance with polymeric additives.

The polymeric ligands on PGNPs are critical for the nucleation and growth of PGNP crystals. In control experiments using oleylamine (OAm) stabilized Au NPs, no ordered 3D crystals were observed (Supplementary Fig. 12). Coassembly of PGNPs and OAm-NPs resulted in crystallization of only the PGNPs, with the OAm-stabilized NPs excluded (Supplementary Fig. 13). The spontaneous phase separation between NPs with two different ligand chemistries confirmed that the PS ligands play an important role in the PGNP crystal growth process. These results support our hypothesis that the polyolefin preferentially precipitates on NPs with polymeric ligands, and thus modulates PGNP nucleation and crystallization.

PGNP crystal growth depends on the solvent evaporation process. Grazing incidence/transmission SAXS (GI(T)SAXS) was performed to monitor the growth of PGNPs on a Si substrate during solvent evaporation. As a transmission technique, GI(T)SAXS probes entire assemblies. The initial ${\varnothing }_{{\mathrm{PP}}/{\mathrm{PE}}}$ was set to $0.15 \%$. The final scattering patterns of the dried samples are shown (Fig. 2c). At a solvent evaporation rate of ~1μL min^−1^, diffraction spots appeared at a beam incidence angle of $\alpha =0.8^\circ$. The peaks are assigned using the diffraction patterns of a bcc crystal lattice with zone axes of [111], [100], [011], and [311] (Supplementary Fig. 14). The presence of defined peaks indicates that, throughout the whole sample, the bcc crystals formed with (110) planes aligned parallel to the substrate (Fig. 2d). Thus, the crystals formed during solvent evaporation are strongly biased by the substrates due to the low cohesive energy of PGNP clusters^30^. There was no diffraction spot when $\alpha =0.14^\circ$ (Supplementary Fig. 15), meaning PGNP crystals were not formed directly at the Si substrate/PGNP interfaces^31^, again differentiating with the previously reported NP interfacial assembly mechanism^10,11,24^.

When the solvent evaporation rate was increased to 2.5 μL min^−1^, very few ordered assemblies formed, indicating that the PGNPs did not have enough mobility to organize into crystals. On the other hand, when the solvent evaporation rate was decreased to 0.7 μL min^−1^, a combination of substrate-oriented crystals, 2D layers, and randomly oriented crystals formed (Supplementary Fig. 16), and the resulting scattering patterns were a combination of diffraction spots and vertical lines. Layered superlattices are typically found when the PGNPs assemble at interfaces without polyolefins. They are formed due to capillary forces^32^ and preferential lateral diffusion. As the PGNP flux was low^33^ at low solvent evaporation rates, PGNPs close to the solvent drying front were able to stack at the droplet-substrate interfaces to form 2D layers, while others continued to grow into 3D PGNP crystals. As the PP/PE loading was increased, however, the precipitant overwhelmed the substrate effect, and randomly oriented crystals formed. A phase diagram is plotted to map out the morphologies formed with varying amounts of polymeric additives and solvent evaporation rates (Fig. 2e).

With a controlled PP/PE amount and solvent evaporation rate, single-crystalline superlattice clusters with preferred orientation formed exclusively upon solvent evaporation (<20 min). Single crystals of (5.3k) Au-PS_0.60_ NPs formed at ${\varnothing }_{{\mathrm{PP}}/{\mathrm{PE}}}$ ~ $0.1 \%$ with a solvent evaporation rate of 0.8 μL min^−1^. Multiple TEM images are provided to demonstrate the high-yield crystal formation. Each of the crystals has distinctive rod-like shape and clear bcc packing, supported by the diffraction spots observed in fast Fourier transforms of the TEM images (Supplementary Fig. 17).

### Electron tomography and reconstruction on a single PGNP crystal

**Fig. 3:**
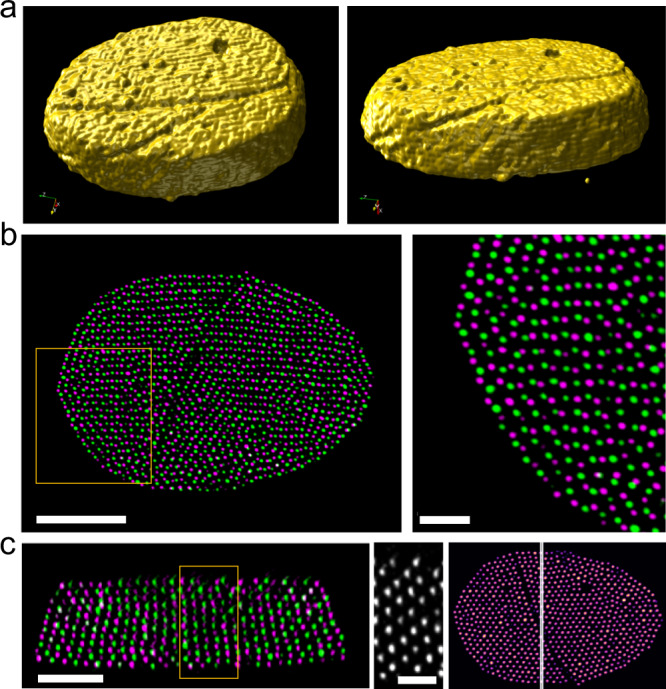
Electron tomography and reconstruction of a single bcc crystal composed of (5.3k) Au-PS_0. 60_ NPs on the substrate. PP/PE was added at ${\varnothing }_{{\mathrm{PP}}/{\mathrm{PE}}} \sim 0.1 \% .$ **a** 3D visualization of the crystal surface (see “Methods”) clearly illustrating its overall shape and the defective top layers. **b** Two base plane layers plotted in green and purple, demonstrating a distorted hexagonal lattice in each plane. The registration between the layers is predominantly bridge sites, as seen in the enlarged view on the right. Scale bars are 100 and 25 nm. **c** Two consecutive planes within a vertical slice plotted in green and purple. PGNPs in one plane sit at the center of the PGNP squares in the other plane. The square lattice of one of the bcc (001) planes is shown in the enlarged view in the middle. The image on the right shows where the cut lies in the top view. Scale bars are 50 and 25 nm.

Scanning transmission electron microscopy (STEM) tomography was performed to characterize the 3D spatial organization of the PGNPs in a single crystal formed on a carbon substrate^34^. The reconstruction method and additional details can be found in the “Methods” section. The reconstructed cluster shows a 3D disk shape composed of stacked PGNP layers. A slight decrease in the number of PGNPs in each layer, from the bottom to the top, is observed. Within the reconstructed crystal, the layers closer to the top and the bottom (substrate) planes have more defects and grain boundaries (Fig. 3a). Figure 3b shows the two base plane layers plotted in different colors, demonstrating a locally distorted hexagonal lattice in each plane. The registration between the layers is predominantly bridge sites, and thus is consistent with bcc stacking and the base plane is (110). The in-plane orientation, i.e., the (001) direction, is mostly horizontal as demonstrated, but varies close to the crystal edge. As shown in the enlarged area in Fig. 3b, the orientation is rotated by roughly 60° or −60°. Similar registration is also seen in base planes near the top, where a hollow site stacking can be observed near the edge (Supplementary Fig. 18). PGNP organization in these regions thus probably reflects the structural transition from hexagonal packing to a bcc lattice during crystal growth. The fact that such organization is more prominent at edges and top layers indicates that the crystal grows from the center to the edges. Out-of-plane structure is examined by analyzing PGNP stacking in the vertical direction. Figure 3c is a slice cut that lies vertically in the crystal with two consecutive PGNP layers depicted in different colors. The figure on the right shows the slice cut from a top-down view. The layers are identified as bcc (001) planes to reflect the majority orientation. Their horizontals are the [−110] direction and their verticals are the [110] direction. The image in the middle depicts a projection of one of the layers, which clearly shows the square lattice of the bcc (001) plane. Based on the structural characterization, we can confirm that the structure of crystals formed on carbon substrates is bcc, and thus identical to the structure of crystals formed in solution. The crystal orientation is biased by the surface yielding (110) base planes. The defects present on the crystal’s surface and its rounded shape reveal that the crystal growth is limited by PGNP diffusion at the late stage of the solvent evaporation. This is not unexpected, considering the drastic viscosity change associated with increasing ${\varnothing }_{{\mathrm{PP}}/{\mathrm{PE}}}$. However, it opens up the possibilities to produce faceted crystals rapidly since cohesive energy density and crystal growth kinetics can be tuned independently.

### Pattern PGNP crystals on substrates

**Fig. 4:**
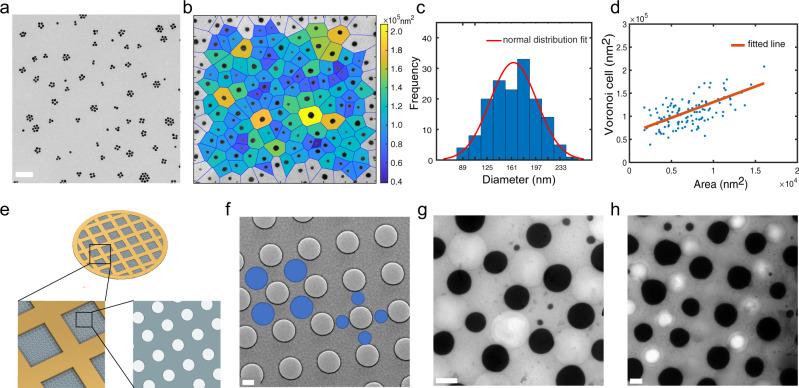
Controlled PGNP crystallization on substrates. **a** PGNP clusters assembled from 25 nm Au NPs (PS = 6k) with PP/PE at ${\varnothing }_{{\mathrm{PP}}/{\mathrm{PE}}}$ ~ $0.05 \%$. Scale bar is 200 nm. **b** TEM image of (1.7k) Au-PS_0.70_ NP crystals formed with PP/PE at ${\varnothing }_{{\mathrm{PP}}/{\mathrm{PE}}}$ ~ $0.08 \%$ and Voronoi representation color coded by the Voronoi cell area in 2D. **c** Crystal size distribution plot, fitted with a normal distribution curve. **d** Voronoi cell area vs. crystal size in 2D, fitted with a linear curve. (1.7k) Au-PS_0.70_ NP crystals characterized in **c** and **d** were formed with PP/PE at ${\varnothing }_{{\mathrm{PP}}/{\mathrm{PE}}}$ ~ $0.08 \%$. **e** Schematic illustration of the templated carbon films with orthogonally arrayed holes mounted on TEM grids. Two possible nucleation sites are highlighted with blue circles in **f**. **g** (1.7k) Au-PS_0.70_ NP crystals formed at bridge sites between adjacent holes. **h** (1.7k) Au-PS_0.70_ NP crystals formed at center sites of the hole lattice. PP/PE was added at ${\varnothing }_{{\mathrm{PP}}/{\mathrm{PE}}} \sim 0.15 \%$. Scale bars are 500 nm in **f**–**h**.

PGNP crystal growth is most readily controlled via the solvent evaporation rate, but crystallization kinetics can also be modulated by changing the NP size. When 25 nm PGNPs were used, individual small clusters were found at ${\varnothing }_{{\mathrm{PP}}/{\mathrm{PE}}}$ ~ $0.05 \%$ (Fig. 4a). Decreased cluster size is attributed to the decreased diffusivity of larger particles according to the Stokes–Einstein equation: $D=\frac{{k}_{B}T}{6\pi \eta r}$ (1), where $\eta$ is the dynamic viscosity and $r$ is the radius of a spherical particle. Dimers, trimers, tetramers, pentamers, and hexamers were all observed (Supplementary Fig. 19). Such geometries of metal nanostructures have received great attention in the past years because of their unique applications in photocatalysis, surface-enhanced spectroscopies, and nonlinear optics^35^. Previously, specific clustering of two to six PGNPs has been a major challenge and was only realized through elaborate templating strategies^36–39^. It is worthwhile to further explore the possibilities of separating these nanostructures with high purity.

Low-magnification TEM images demonstrate that the growth of 3D crystals is not a rare event and is highly reproducible. At ${\varnothing }_{{\mathrm{PP}}/{\mathrm{PE}}} \sim 0.05 \%$, the sizes of the crystals can be fitted to a normal distribution with an average diameter of ~165 nm. The crystals are approximated as disks, and their sizes are described by diameter $d=2\sqrt{{\mathrm{area}}/\pi }$ (2). TEM images were further analyzed by Voronoi tessellation to visualize the corresponding occupied area of each cluster. Figure 4b shows the 2D map of Voronoi cells, which are color coded based on their area. The nucleation sites were randomly distributed. Fitting the linear model to the Voronoi cell area vs. the size of the corresponding crystals gives a slope of (6.8 ± 1.9) and an intercept of (6.3 ± 1.4) × 10^4^ nm^2^ (Fig. 4d). The linear correlation between the two suggests that a 2D self-assembly model can be applied^11^. Once a nucleus forms, the surrounding area is depleted of PGNPs, and further growth of the crystal relies on the short-range diffusion and local rearrangement of PGNPs.

Furthermore, the capability to control the PGNP nucleation and growth process is promising for producing hierarchical assemblies^40–42^. Patterned surfaces can be used as templates to control the location of NP arrays, taking advantage of various interactions between the NPs and substrates. As an example, PGNPs can be assembled into a square array of crystals by using templated carbon films (Fig. 4e). The commercially available carbon-coated TEM grids have orthogonally patterned vacuum holes of ~1 μm diameter and ~650 nm separation. The available spaces between the holes are comparable to the size of PGNP crystals formed. Here, the nucleation of PGNPs preferentially took place between the holes, and the crystals were thus organized into a square lattice (Fig. 4g, h). Crystallinity was maintained within individual clusters (Supplementary Fig. 20). At least two periodicities existed: the several nanometer inter-PGNP distance in each cluster, and the micrometer separation between clusters. Within the patterned substrates, the PGNPs had two possible nucleation sites: either between two adjacent vacancies or at interstitial sites of the hole lattice (see blue dots in Fig. 4f). These two sites produced two distinct separation periodicities and cluster sizes, due to the difference in available space. The average diameters of the crystals were ~600 and ~900 nm, respectively.

We demonstrated that a properly chosen polymer precipitate and PGNP surface modification can effectively change NP/solvent interactions, leading to controlled nucleation of PGNPs in dilute solutions. The initial PGNP clustering occurs in solution due to the precipitation of poorly soluble polymers onto PGNP surfaces. The crystallization process can be further modulated by manipulating the solvent evaporation rate, NP size, and nucleation sites. These PGNP systems are ideal for future studies in crystallization at single-building-block level, since the NP assembly’s energetic driving force, kinetics, and pathways can be easily modulated. The growth of NP superlattice crystals within several minutes, as demonstrated here, removes an important roadblock toward NP-based composite materials. Lastly, these PGNP crystals are obtained with the presence of trace amount of impurities from plastic containers that are routinely used. The results shown here clearly underscore the importance of identifying any potential chemical impurities and their effects in the field of nanoscience, even at a trace amount.

## Methods

### Materials

Hydrogen tetrachloroaurate (III) trihydrate (HAuCl_4_•3H_2_O, Sigma-Aldrich, 99.995%, trace metals basis), OAm (Sigma-Aldrich, technical grade, 70%), tert-butylamine-borane complex (Aldrich, 97%), 1,2,3,4-tetrahydronaphthalene (tetralin, anhydrous, 99%), styrene (ReagentPlus^®^, contains 4-tert-butylcatechol as stabilizer, ≥99%), 2-Cyano-2-propyl dodecyl trithiocarbonate (chain transfer agent) (Sigma-Aldrich, >97% (HPLC)), 2,2′-Azobis(2-methylpropionitrile) (AIBN) (Sigma-Aldrich, 98%), hydrazine (Sigma-Aldrich, anhydrous, 98%), and toluene (Sigma-Aldrich, ACS reagent, ≥99.5%) were used in this work.

Polyolefin mixtures were dissolved from VWR high-performance microcentrifuge tubes, PP, 1.5 mL. PP/PE stock solution was prepared by immersing a microcentrifuge tube into 10 mL toluene for 1 week and dissolved polyolefin was weighted after removing the solvent.

Carboxylic acid terminated poly(N-isopropylacrylamide) (PNIPAM-COOH, Mn = 6.3 kg mol^−1^, PDI = 1.4), carboxylic acid terminated polybutadiene (PBd-COOH, Mn = 2.5 kg mol^−1^, PDI = 1.3), and PS-b-poly(cholesteryloxycarbonyloxy ethyl methacrylate) (PS-b-PHEMAC, Mn = 29 kg mol^−1^ for PS, 28 kg mol^−1^ for PHEMAC, PDI = 1.06) were purchased from Polymer Source Inc.

### Synthesis of PGNPs

3.8 nm OAm-Au NPs were synthesized according to literature methods^43^. The synthesis was carried out under a nitrogen atmosphere using standard Schlenk line techniques. Thiol-terminated PS (PS-SH) was synthesized using radical addition fragmentation transfer polymerization and end-functionalized by aminolysis. Au NPs grafted with PS-SH were prepared by a ligand exchange process as reported before^24^. Further details on synthesis and characterization are provided in Supplementary Information.

### PGNP crystal growth upon solvent evaporation

Au PGNPs were dispersed in toluene at ∅_NP_ = 0.1%. Polymer additives were dissolved in toluene and added to the PGNP solution at ∅_polymer_ ranging from 0.01 to 0.5%. Upon solvent evaporation, samples deposited on Si wafers and TEM grids were used for GISAXS measurements and TEM analysis, respectively. For crystal growth on normal substrates, 50 µL of solution was drop-cast on 200 mesh Cu TEM grids or a Si substrate and dried after ~20 min. For a quick-drying experiment, less than 5 µL solution was drop-cast and the solution dried within 1 min. Solvent evaporation was done under ambient conditions. For crystal growth on templated TEM grids, slow solvent evaporation and relatively high polymer concentration (∅_polymer_ ~ 0.15–0.2%) are desired in order to get highly ordered crystals. The TEM grid was laid on a Si substrate and placed in a Teflon well with dimensions of 1.5 cm $\times$ 1.5 cm $\times$ 1.0 cm, and covered with a glass coverslip to slow down the solvent evaporation. Samples dried completely after 2 h.

### Transmission electron microscopy

Au PGNPs and their assemblies were imaged using a FEI Tecnai 12 at an accelerating voltage of 120 kV.

### Small-angel X-ray scattering

In situ solution SAXS experiments were performed at Beamline 11-BM (Complex Material Scattering) of National Synchrotron Light Source-II (NSLS-II, Brookhaven National Laboratory). The SAXS data were collected on a Dectris 2M detector at a sample-to-detector distance of 2 m, using an X-ray beam with an energy of 13.5 keV (the corresponding wavelength *λ* = 0.92 Å). Au PGNP solution was positioned so that the beam went through the solution. The solution was left dry under ambient environment and were exposed to X-rays every 30 s. The *q* range is 0.001°–0.2°.

Static SAXS experiments were performed at beamline 7.3.3 at Advanced Light Source in Lawrence Berkeley National Laboratory. The X-ray wavelength is 1.24 Å. The scattered X-ray intensity distribution was detected using a high-speed detector, Pilatus 2M. Images were plotted as intensities (*I*) vs. *q*, where *q* = (4π/*λ*) sin(θ), *λ* is the wavelength of the incident X-ray beam, and 2θ is the scattering angle.

### Peak deconvolution

The scattering peaks were deconvoluted using a MATLAB command-line peak fitting program^44^. The area of interest, containing the sharp diffraction peak at *q* ~ 0.1 Å^−1^ from PGNP crystals and the broad peak from PGNP clusters in the solution, was selected to fit with a two-peak Gaussian model. At least ten trial fits were performed and the one with the lowest fitting error was selected. Data points were collected as the integration ratio of the two deconvoluted peaks.

### Grazing incidence/transmission SAXS

GI(T)SAXS experiments at an incident angle *α* of 0.14°–0.8° were conducted at beamline 8‐ID‐E at the Advanced Photon Source of Argonne National Laboratory. Samples were drop-cast on a 1 cm $\times$ 1 cm Si substrate with 100–500 µL solvent reservoir placed in the house-made solvent annealing chamber. The chamber was sealed and N_2_ flow was purged through to control the solvent evaporation. The scattered X-ray intensity distribution was detected using a high-speed detector, Pilatus 1M. Samples were exposed to 7.35‐keV radiation (*λ* = 1.68 Å).

### STEM tomography

The tomography experiment was performed using a FEI Titan microscope operated at 300 kV. The HAADF-STEM images were acquired with a 10 mrad probe semi-convergence angle. The projections of PGNPs were recorded over the tilt range of −70° to 70°, with 2° step size and pixel size of 0.63 nm. The alignment was performed on IMOD (version 4.9.10), making use of fiducial markers and custom python scripts. Reconstructions were performed using inverse radon transformation from the scikit-image python library.

Figure 3a was rendered as a solid surface whilst conserving the larger defects and morphological features by filling the interparticle gaps. The filled volume was created from the original reconstruction data by simulating the NPs in the lattice as Gaussian peaks and convoluting this data with a spherical kernel.

Thin slices capturing single-layer planes were extracted from the reconstruction volume. As the base plane and other “horizontal” lattice planes of the NP crystal are curved, the slices were cut along curved planes. In the vertical cuts, the thin slices did not follow the curved lattice planes but were planar. Along the thickness of the thin slices either the average or the maximum intensity was determined to yield a 2D intensity map from each thin slice. For the images, a nonlinear contrast function was employed to better accentuate the NP positions in the figures. The two-layer images are composites from two neighboring layers where an image from each layer is generated as discussed above and provides the intensity for its assigned layer color. The identical contrast function is used for both layers.

## Supplementary information

Supplementary Information

## Data Availability

The data that support the findings of this study are available within the paper and its Supplementary Information Files.

## Code availability

Custom MATLAB codes for Voronoi construction are available from the corresponding author upon request. MATLAB package for peak fitting and deconvolution is an open-source package and available online: https://www.mathworks.com/matlabcentral/fileexchange/23611-peakfit-m.
