# Supplementary material for: Crystallization of nanoparticles induced by precipitation of trace polymeric additives
Source: Nat Commun. 2021 May 13;12:2767. doi: 10.1038/s41467-021-22950-2 (PMC8119732; doi:10.1038/s41467-021-22950-2)
Supplement: Supplementary file 1 — Supplementary Information [file 41467_2021_22950_MOESM1_ESM.pdf]

SUPPLEMENTARY INFORMATION of

# Crystallization of nanoparticles induced by precipitation of trace polymeric additives

Yiwen Qian, Alessandra Da Silva, Emmy Yu, Christopher L. Anderson, Yi Liu, Wolfgang Theis, Peter Ercius, and Ting Xu*

*Correspondence to: tingxu@berkeley.edu

**Contents:**

**Supplementary Methods and Discussions**

1. Synthesis and characterization of PGNPs and PGNP assemblies ………………….……2
2. Control experiments ………………………………………………………………………7
3. Characterization of polymer additives ……………………………………………………8
4. *In-situ* SAXS experiments………………………………………………………………13
5. 3D crystals assembled at different PP/PE volume fractions……………………………..14
6. Assemblies of OAm-Au NPs……………………………………………………………..15
7. *In-situ* GI(T)SAXS experiments…………………………...……………………………17
8. PGNP assemblies with different morphologies……………………………………...…..18
9. Individual 3D PGNP crystals……………………………………………………………19
10. Electron tomography and reconstruction ………………………………………………..20
11. PGNP clusters assembled from 25nm Au PGNPS ……………………………………...21
12. PGNP clusters assembled on patterned substrates ………………………………………22

## 1. Synthesis and characterization of PGNPs and PGNP assemblies

*Synthesis of Au NPs(1):*

In a typical synthesis of ~ 4 nm Au NPs, an orange precursor solution of tetralin (10 mL), OAm (10 mL), and $HAuCl_4 \cdot 3H_2O$ (100 mg) was prepared at room temperature (r.t., ~20°C) and degassed three times. The solution was then kept in an ice bath and N2 was purged through. A reducing solution containing 0.5 mmol of TBAB (43 mg), tetralin (1 mL), and OAm (1 mL) was mixed under sonication and quickly injected into the precursor solution. The reduction was instantaneously initiated and the solution changed to a deep purple color within 5 s. The mixture was allowed to react in the ice bath for 1 hour before acetone (60 mL) was added to precipitate the Au NPs. Au NPs were collected by centrifugation (7,200 × g, 10 min), washed with methanol twice, and re-dispersed in toluene. The resulting NPs have the oleylamine as the stabilizing ligand and are denoted as OAm-Au NPs.

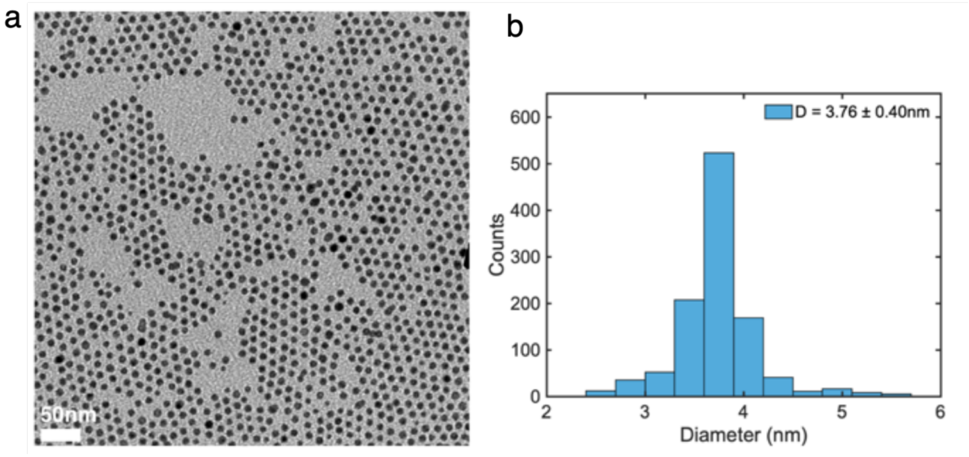


**Supplementary Fig. 1 | TEM image of OAm-Au NPs and NP size distribution histogram.** (a) A representative TEM images of OAm-Au NPs is shown. (b) The size histogram of OAm-NPs. The average NP core size is 3.8 nm

*Synthesis of thiol end-functionalized polystyrene:*

(Radical Addition Fragmentation Transfer) RAFT polymerization was employed to synthesize polystyrene (PS). In a typical synthesis of PS, targeting Mn = 3k and conversion of 60%, styrene monomer, chain transfer agent and AIBN were mixed at a molar ratio of 237.5 : 5 : 1 and degassed

with three freeze-pump-thaw cycles. The mixture solution was then put into a pre-heated 75°C oven until the aliquot became viscous. 2 mL hydrazine mono-hydrate was added to the reaction solution and dissolved using 10 mL THF under vigorous stirring. The thiol-terminated PS was precipitated with 30 mL methanol and was collected by centrifugation (7,200 × g, 5 min). The purification procedure was repeated three times to remove the unreacted monomers and initiators. The final product was dried in vacuum oven at r.t. over-night. Styrene monomer was purified by distillation to remove the inhibitor prior to the synthesis. AIBN was recrystallized before use.

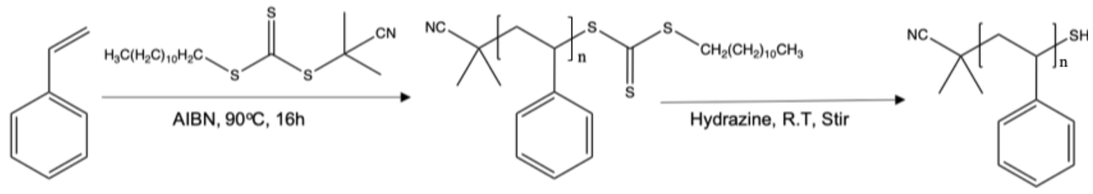


*PS characterization:*

$^1$H nuclear magnetic resonance (NMR) spectra were obtained with a Bruker Advance 400 spectrometer (400 MHz) using a 5-mm *Z*-gradient broadband observe probe. PS molecular weight was determined using gel permeation chromatography with an Agilent 1260 Infinity series instrument equipped with two Agilent PolyPore columns (300 mm × 7.5 mm), calibrated using PS standards. The runs were performed with tetrahydrofuran as mobile phase at 1 mL min$^{-1}$.

*Thiol Functionalization of OAm-Au NPs(2):*

A toluene solution of OAm-Au NPs with a NP concentration of 5 mg mL$^{-1}$ was mixed with a 40 mg mL$^{-1}$ PS-SH solution under sonication. The mixture solution was stirred vigorously for 3 days. The PS-modified Au NPs were collected by methanol precipitation and centrifugation at 7,200 × g. for 3 min. The supernatant was removed and the PGNPs were redispersed in toluene. The above solvent–nonsolvent cleaning procedure was repeated at least 6 times to remove excess unbound PS.

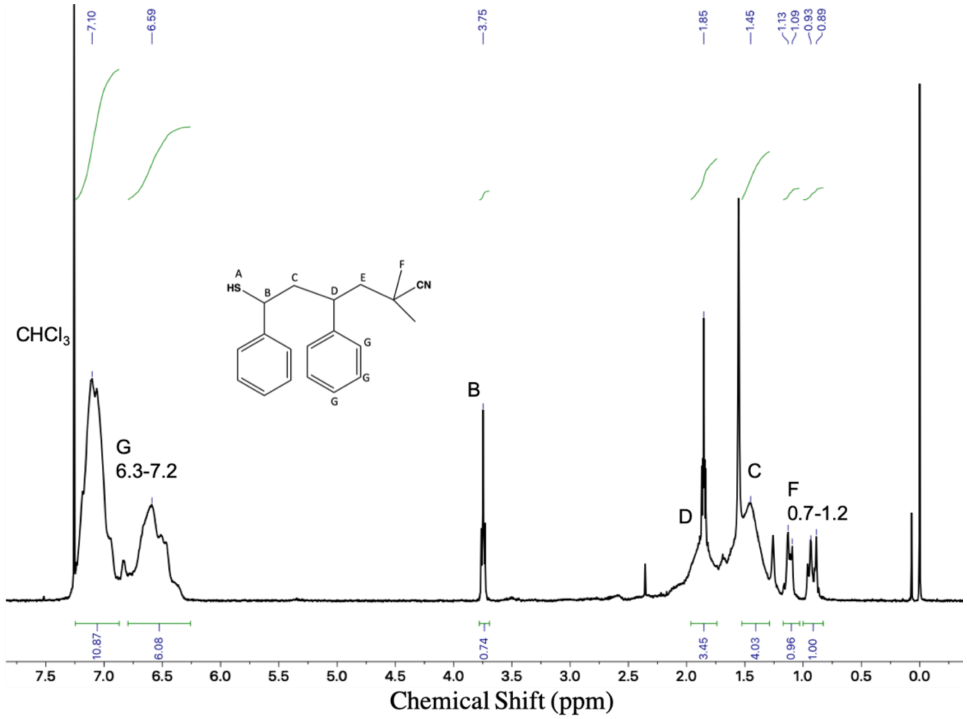


**Supplementary Fig. 2 | $^{1}$H-NMR spectrum of PS (Mn = 1.7k)** (CDCl3, 400MHz), δ(ppm): 7.10 (br), 6.59 (br), 3.75 (m), 1.85 (m), 1.45 (br), 1.09-1.13 (br), 0.93-0.89 (br).

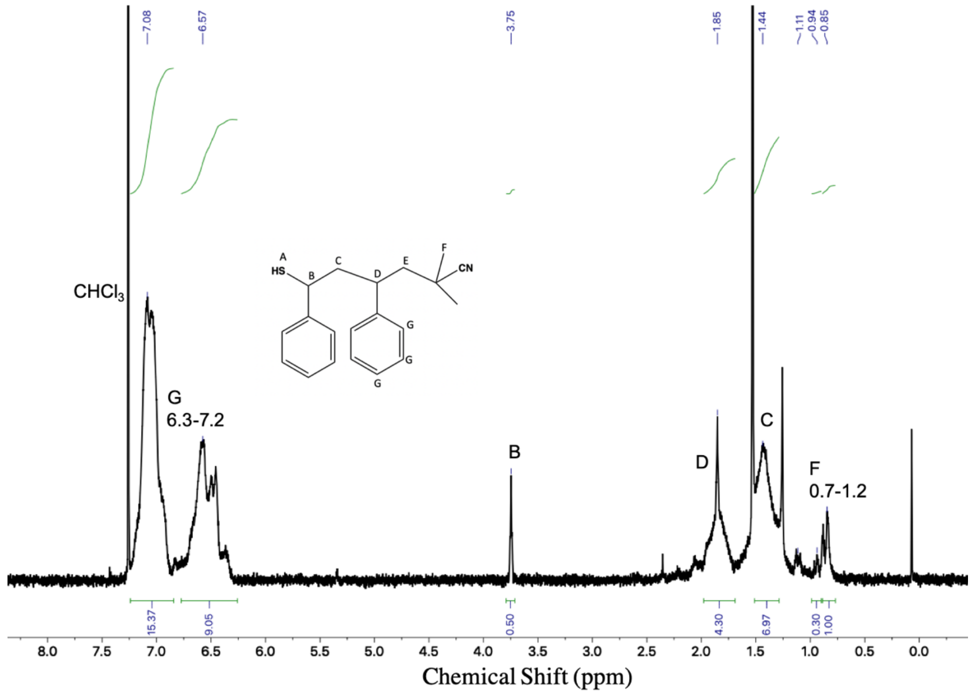


**Supplementary Fig. 3 | $^{1}$H-NMR spectrum of PS (Mn = 5.3k)** (CDCl3, 400MHz), δ(ppm): 7.08 (br), 6.57 (br), 3.75 (m), 1.85 (m), 1.44 (br), 1.11 (br), 0.94-0.85 (br).

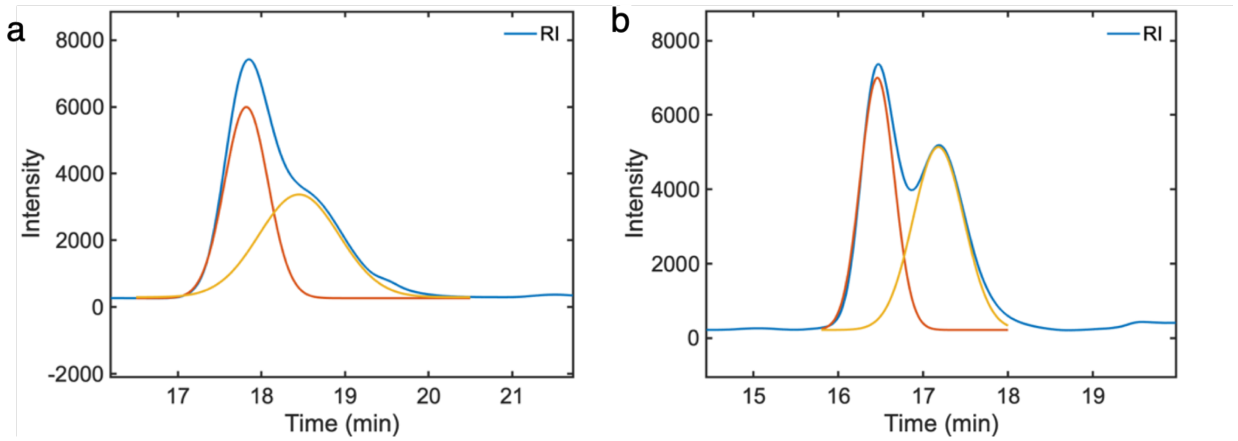


**Supplementary Fig. 4 | GPC traces of PS-SH .** THF-GPC of (a) PS-SH (1.7k) and (b) PS-SH (5.3k). Note the appearance of a small second peak in the GPC trace at a molecular weight of double the main peak. The polymer was coupled to a high molecular weight product with internal disulfide bridges, that retains the ability to attach to the Au surface.

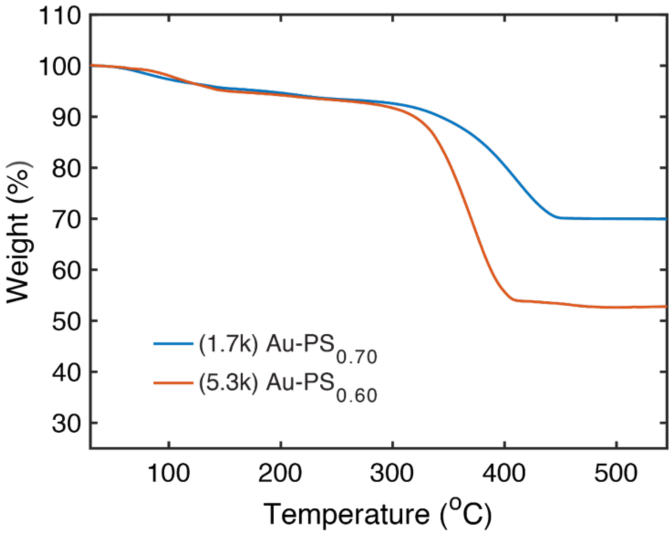


**Supplementary Fig. 5 | TGA characterization on PGNPs.** The TGA traces of different PGNPs are shown. The grafting density is presented in Supplementary Table 1.

**Supplementary Table 1. Characterization results for different PGNPs.**

| | (1.7k) Au-$PS_{0.70}$ | (5.3k) Au-$PS_{0.60}$ |
|---|---|---|
| Mn of PS (g $mol^{-1}$) | 1,680 | 5,300 |
| $\sigma$ (chains $nm^{-2}$) | 0.70 | 0.59 |
| $D$ by FFT (nm) | $6.08 \pm 0.42$ | 7.98 $\pm$0.58 |
| $D$ by scattering (nm) | 6.1 | 8.06 |
| $l$ by FFT (nm) | 1.14 | 2.09 |
| $l$ by scattering (nm) | 1.15 | 2.13 |

Ligand grafting density ($\sigma$) was calculated(8) based on thermogravimetric analysis (TGA) data (**Supplementary Fig. 5**). Average nearest-neighbor center-to-center interparticle distance ($D$) is determined by FFT on TEM images of assembled PGNP crystals. Average ligand shell thickness ($l$) is calculated with $l = (D - d)/2$, $d$ is the particle core diameter. Standard deviation is calculated based on image analyses on at least 3 different sample areas. $D$ and $l$ based on X-ray scattering data are listed as well.

## 2. Control experiments

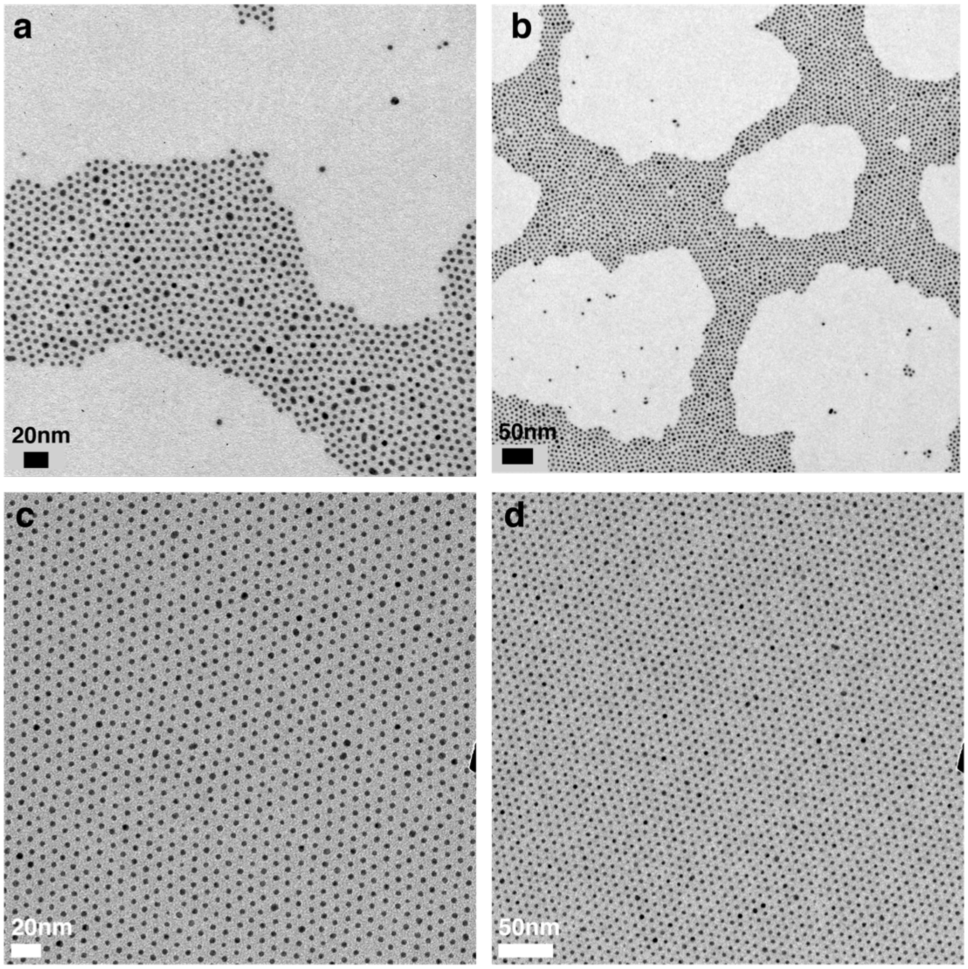


**Supplementary Fig. 6 | 2D assemblies of PGNPs by drop casting on a TEM grid without PP/PE.** Assemblies were composed of (1.7k) Au-$PS_{0.70}$ NPs in (a) and (b) and (5.3k) Au-$PS_{0.60}$ NPs in (c) and (d).

## 3. Characterization of polymer additives

*Methods:*

$^{1}$H nuclear magnetic resonance (NMR) spectra were obtained with a Bruker Advance 400 spectrometer (400 MHz) using a 5-mm *Z*-gradient broadband observe probe. Fourier Transform Infrared (FTIR) spectra were acquired with a Bruker Vertex 80 Time-resolved FTIR spectrometer. Molecular weight distribution of the PP/PE mixture was determined using high temperature size exclusion chromatography (Tosoh EcoSEC HT) with three TSKgel GMHhr-H(S) HT columns in series. The runs were performed with 1,2,4-trichlorobenzene containing 500 ppm butylated hydroxytoluene at 135 °C as mobile phase at 1 mL min$^{-1}$. Relative molecular weight was calculated relative to narrow poly(styrene) standards. GIWAXS experiments at an incident angle of 0.18° were performed at beamline 7.3.3 at Advanced Light Source in Lawrence Berkeley National Laboratory. Images were plotted as intensities (I) vs q, where $q = (4\pi/\lambda) \sin(\theta)$, $\lambda$ is the wavelength of the incident X-ray beam, and $2\theta$ is the scattering angle. Polymer samples were deposited on a 1 cm × 1 cm silicon substrate by drop casting.

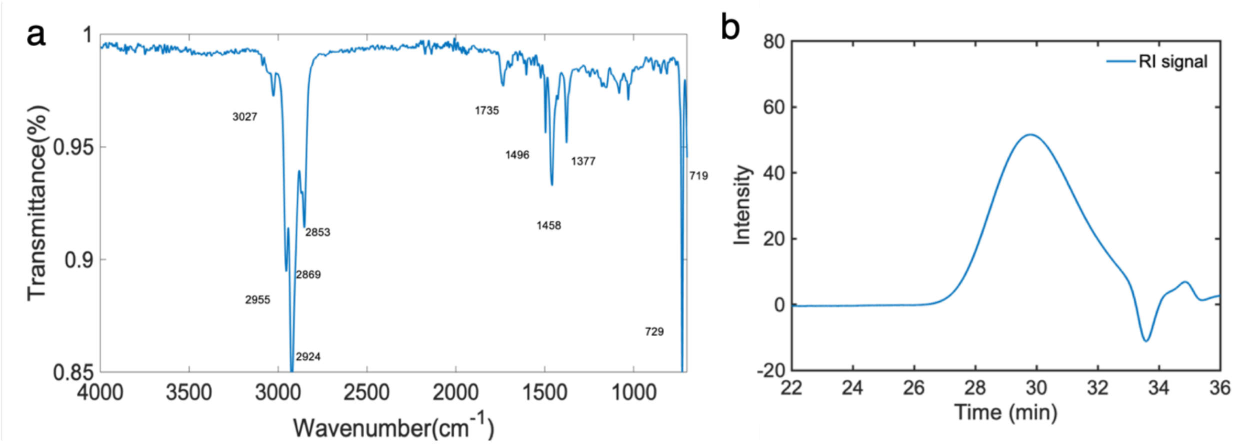


**Supplementary Fig. 7 | Characterizations of PP/PE.** (a) FTIR spectrum of PP/PE. (b) High-temperature SEC trace of PP/PE dissolved in 1,2,4-trichlorobenzene. Mn = 786 kg/mol, Mw = 5,277 kg/mol, PDI = 6.72.

*PGNP assembly with different polyolefin additives:*

In order to understand how chemical composition, molecular weight and crystallinity influence the effects of inducing crystallization, different polyolefins including amorphous PP, isotactic PP, PP/PE, $PP/PE_2$, $PE_1$ and $PE_2$ were examined. Amorphous, isotactic PP, 2 different molecular weight PE ($PE_1$ and $PE_2$) were used as received. $PP/PE_2$ is similar to PP/PE used elsewhere but is instead dissolved from a VWR microcentrifuge tube of 0.5 mL. NMR analysis shows that the two PP/PE samples have a large amount of PE as indicated by the increased intensity of the peak for the $CH_2$ group (1.26 ppm) (**Supplementary Fig. 8)**. The molecular weight of the precipitants also affected the formation of PGNP assemblies. As shown in **Supplementary Table 2**, low Mn polyolefins modulate the interfacial interactions more effectively, due to their small footprints on the PGNP surfaces. Grazing incidence wide-angle X-ray scattering (GIWAXS) shows that PP/PE samples are mainly amorphous (**Supplementary Fig. 9**). Polyolefins with high crystallinity are not preferred since they are more likely to precipitate by themselves rather than interacting with the PGNPs. Thus, PP/PE as an amorphous mixture of PP and PE with low Mn is the most effective precipitant to induce crystallization of PGNPs.

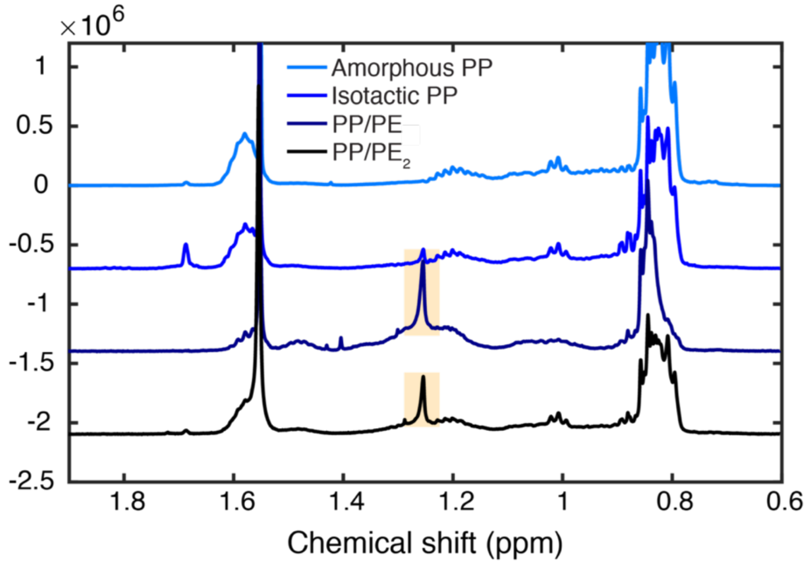


**Supplementary Fig. 8 | $^1$H-NMR spectrum of different polyolefin samples ($CDCl_3$, 400MHz).** Polyethylene $CH_2$ protons are highlighted.

**Supplementary Table 2. Characterization results for different polyolefin additives.** TEM images of the self-assembled (1.7k) Au-$PS_{0.70}$ NPs with the addition of different polyolefins at volume fraction $\varnothing_{polyolefin} \sim 0.1\%$ were shown. Scale bars, 50 nm.

| | Mn (g/mol) | Mw (g/mol) | PE amount indicated by NMR | PGNP assemblies with the addition of polyolefin |
|---|---|---|---|---|
| Amorphous PP | / | / | trace | 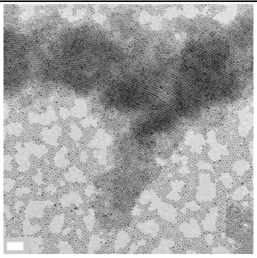 |
| Isotactic PP | 5,000 | 12,000 | trace | 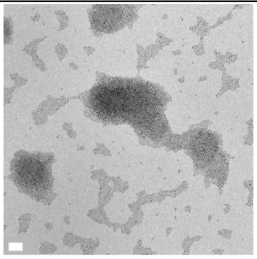 |
| PP/PE | 786 | 5,277 | ~ 10% | 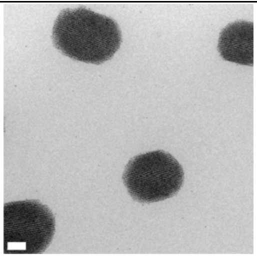 |
| PP/$PE_2$ | 549 | 5,380 | ~ 4% | 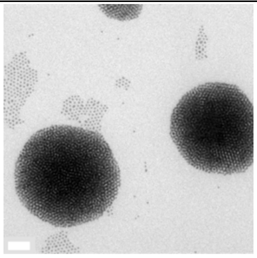 |

| | | | | |
|---|---|---|---|---|
| $PE_1$ | 1,700 | 4,000 | NA | 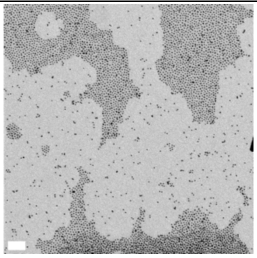 |
| $PE_2$ | 7,700 | 35,000 | NA | 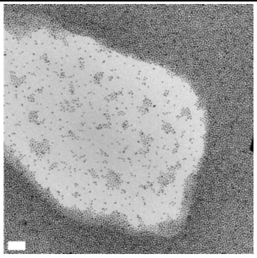 |

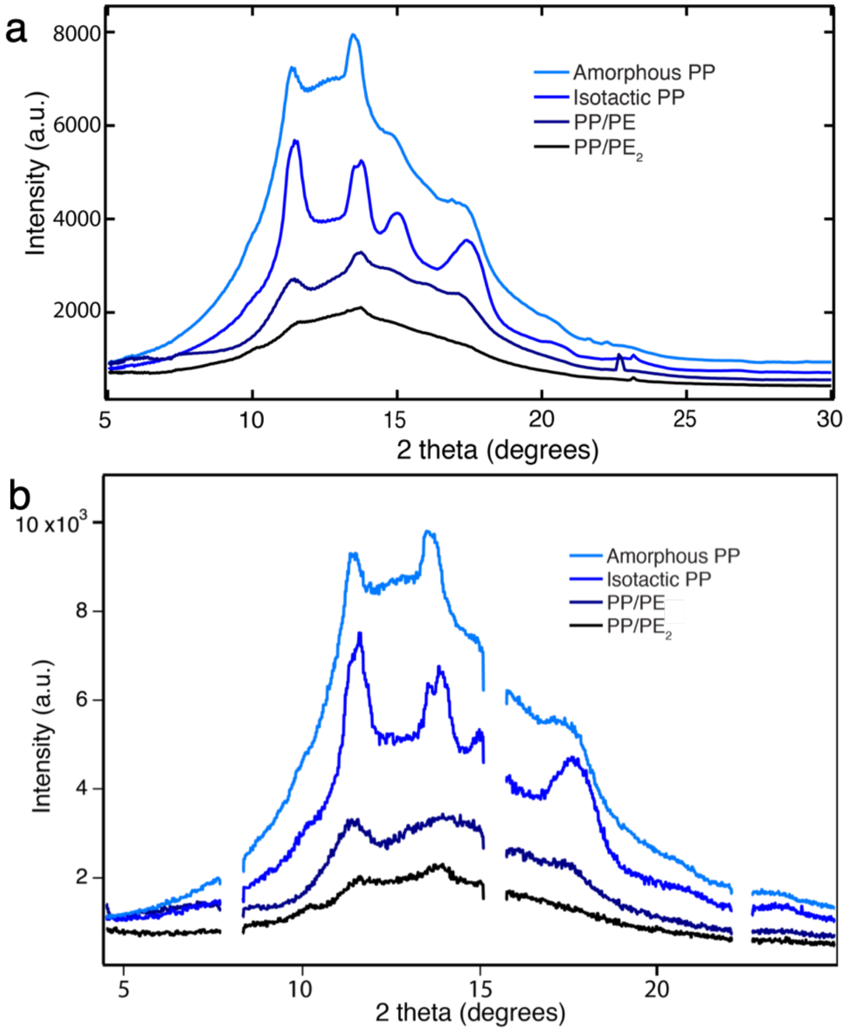


**Supplementary Fig. 9 | One-dimensional radially averaged GIWAXS curves of polyolefins.** GIWAXS curves (a) before and (b) after missing wedge correction. Crystallinity of precipitates was studied by the GIWAXS of the drying polyolefin solution. PP/PE samples are mainly amorphous.

## 4. *In-situ* SAXS experiments

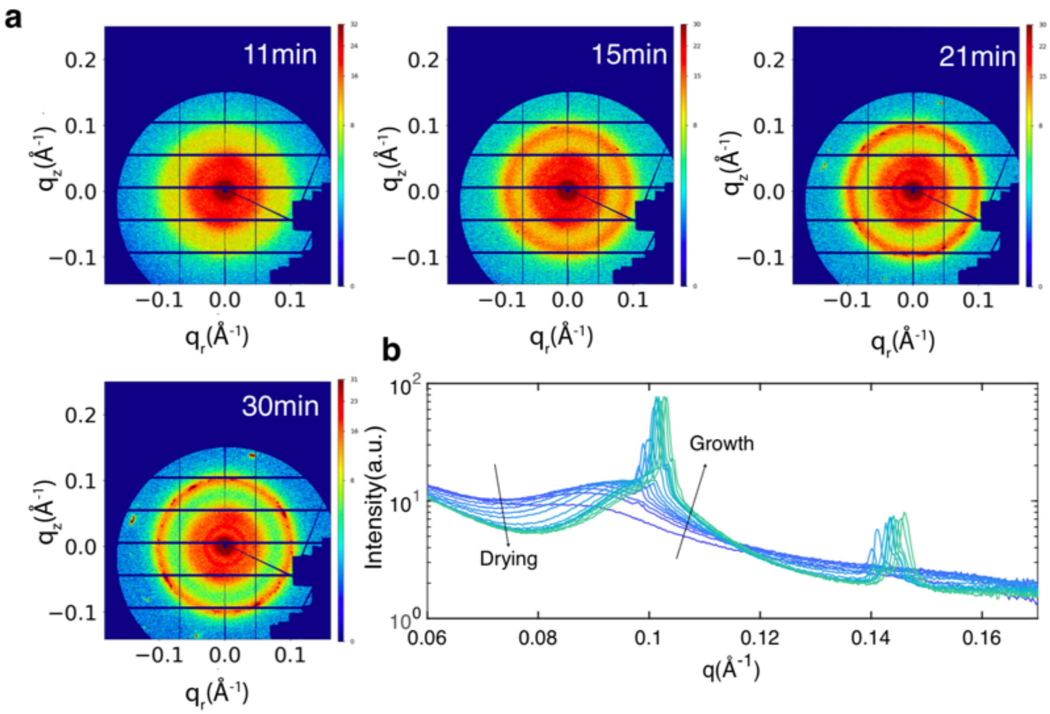


**Supplementary Fig. 10 | Complementary *in-situ* SAXS profiles of Figure 1b.** (a) 2D SAXS images at specified time points. (b) Radial integration of intensity during drying process. Absolute intensity changes with the increasing PGNP concentration and the growth of crystals.

## 5. 3D crystals assembled at different PP/PE volume fractions.

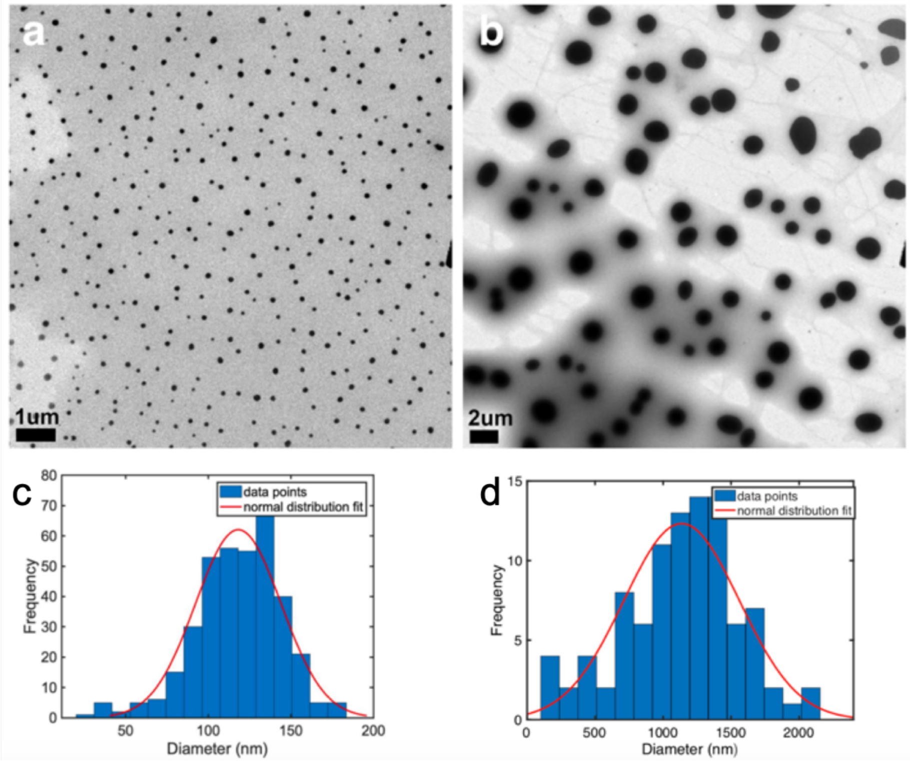


**Supplementary Fig. 11 | 3D crystals assembled from (1.7k) Au-PS0.70 NPs at different $\emptyset_{PP/PE}$.** TEM images of crystals formed with (a) 0.05% and (b) 0.5% PP/PE. Size distribution histograms in (c) and (d) are obtained from TEM image analysis, corresponding to $\emptyset_{PP/PE} \sim 0.05\%$ and 0.5% . The red lines represent the Gaussian fit to the size histograms.

## 6. Assemblies of OAm-Au NPs

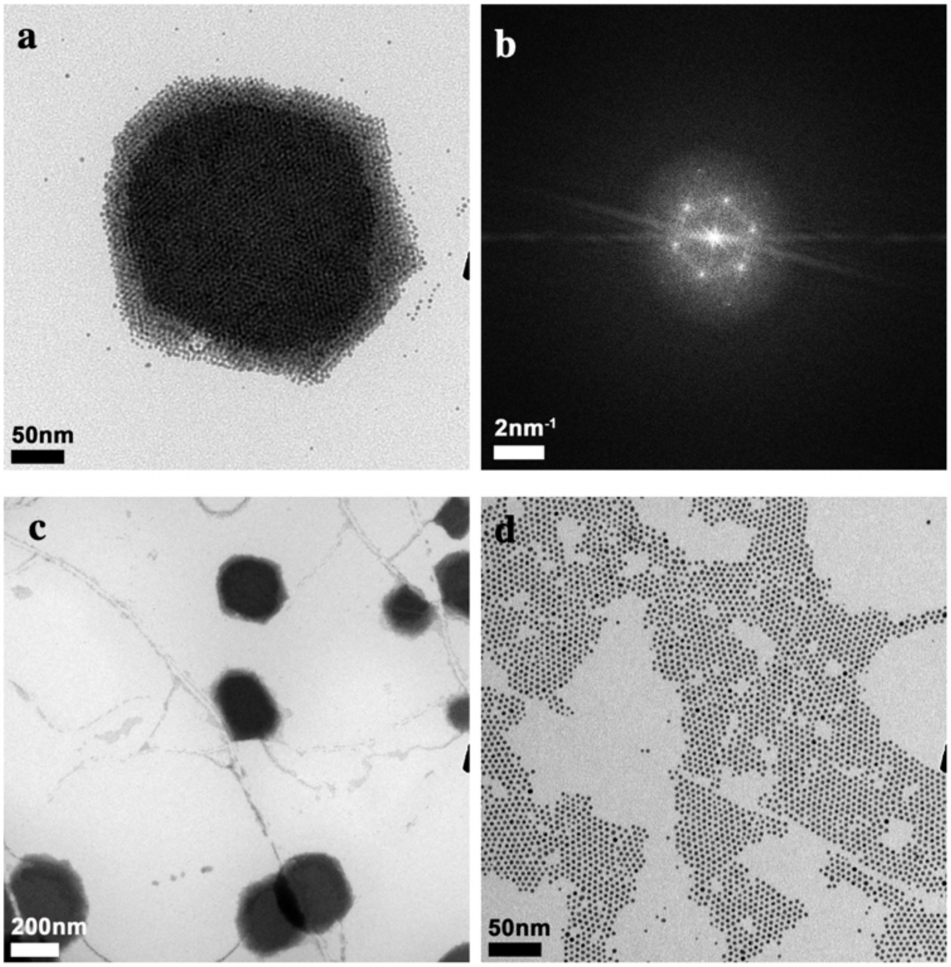


**Supplementary Fig. 12 | OAm-Au NPs assembled with 0.5% PP/PE.** (a) TEM image of A poorly ordered cluster with its corresponding FFT image (b). (c-d) Other areas of OAm-Au NP assemblies.

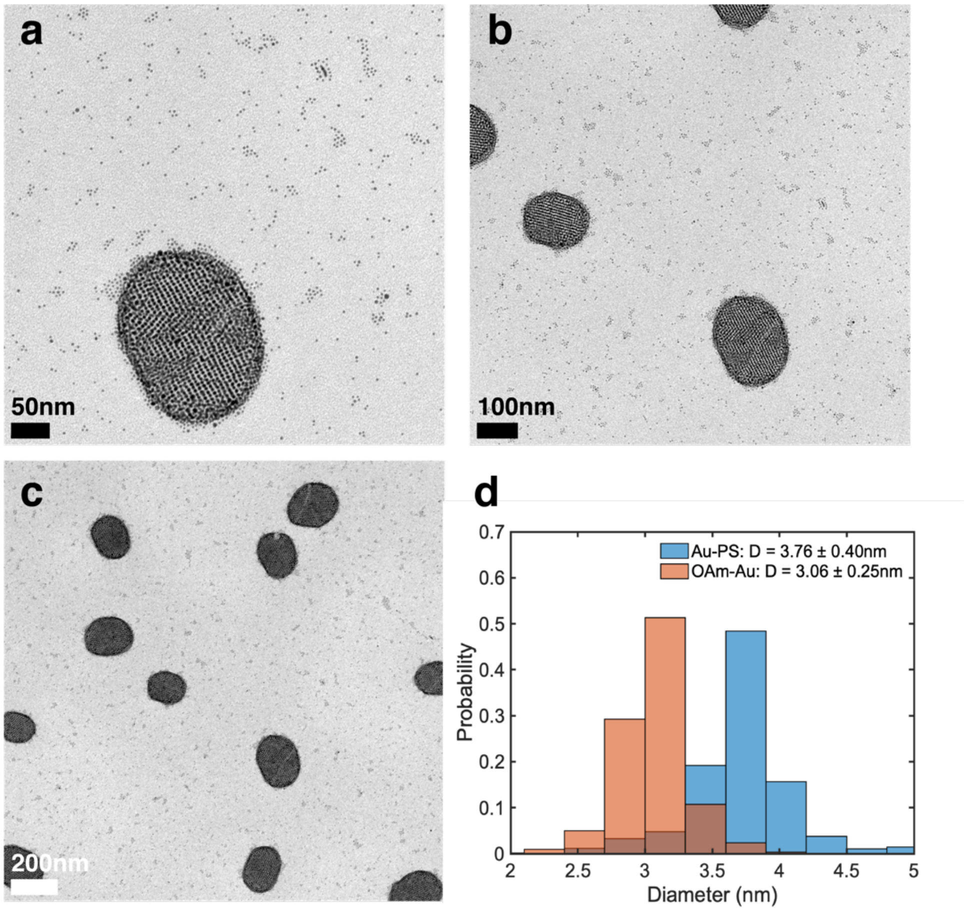


**Supplementary Fig. 13 | Co-assembly of OAm-Au NPs and (1.7k) Au-$PS_{0.70}$ NPs with the presence of PP/PE.** (a-c) Phase separation between OAm-Au NPs (3nm core size) and PS modified Au NPs (3.8nm core size) is manifested by the size differences between the two types of particles. Only PS-Au NPs are assembled into ordered crystals. (d) Size distributions of OAm-Au NPs and Au-PS NPs are plotted.

## 7. *In-situ* GI(T)SAXS experiments

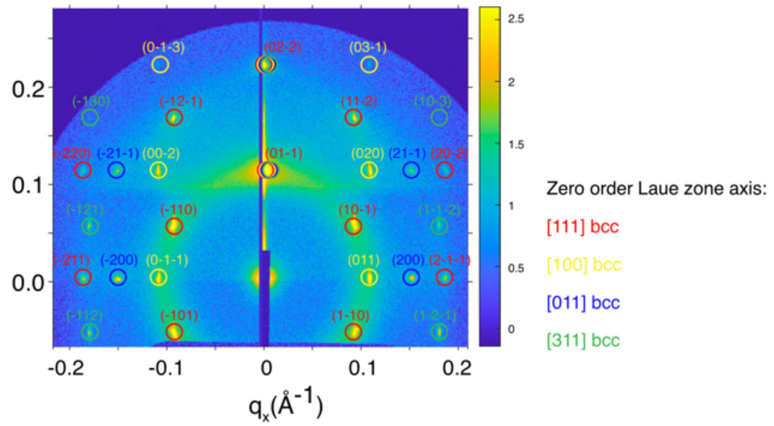


**Supplementary Fig. 14 | GTSAXS diffraction pattern of (1.7k) Au-PS$_{0.70}$ NPs with assigned bcc crystal planes.** The scattering spots are assigned to different sets of diffraction peaks with corresponding zero order Laue zone axes.

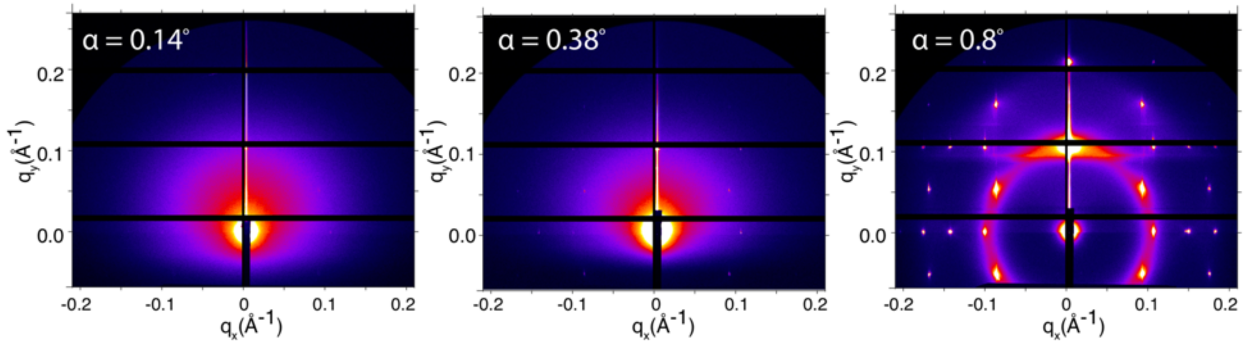


**Supplementary Fig. 15 | GI(T)SAXS patterns of (1.7k) Au-PS$_{0.70}$ NP assemblies at different incident angles.** The GI(T)SAXS patterns are from the same sample position and with the same exposure time.

## 8. PGNP assemblies with different morphologies

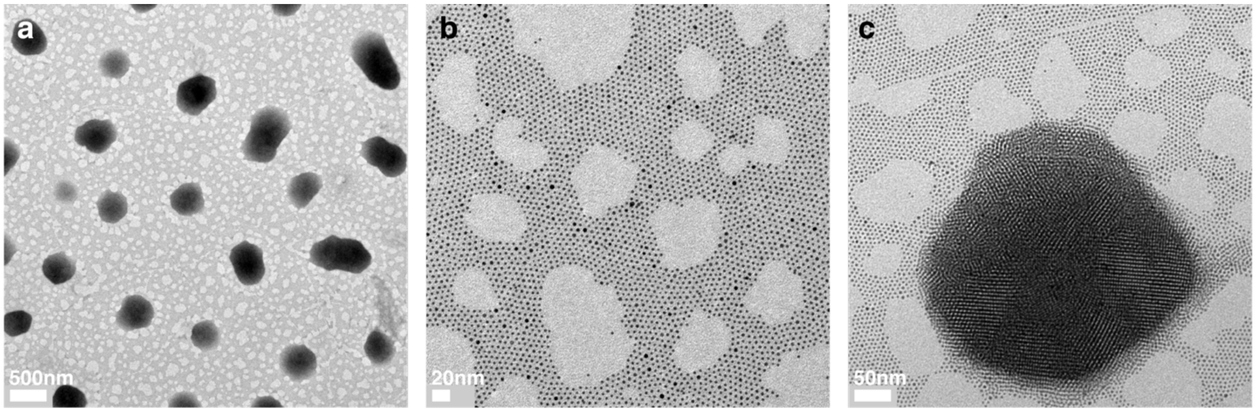


**Supplementary Fig. 16 | TEM images of (1.7k) Au-PS$_{0.70}$ NP assemblies formed at the evaporation rate of 0.7 μL/min.** (a) The assemblies of (1.7k) Au-PS$_{0.70}$ NPs formed with PP/PE. Surface-oriented crystals, 2D layers (b), and randomly oriented crystals (c) are formed.

## 9. Individual 3D PGNP crystals

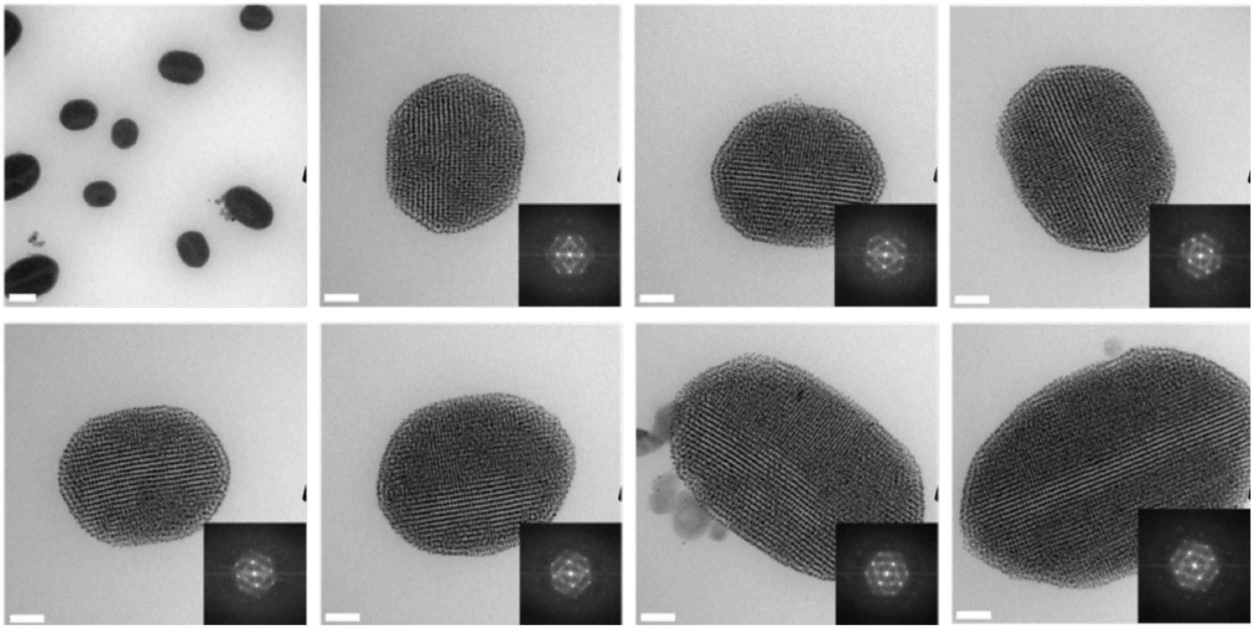

**Supplementary Fig. 17 | Individual crystals formed with the presence of PP/PE.** TEM and FFT images of 3D crystals assembled from Au-$PS_{0.60}$ NPs are shown. Scale bar is 200 nm in the first image and 50 nm in the others.

## 10. Electron tomography and reconstruction

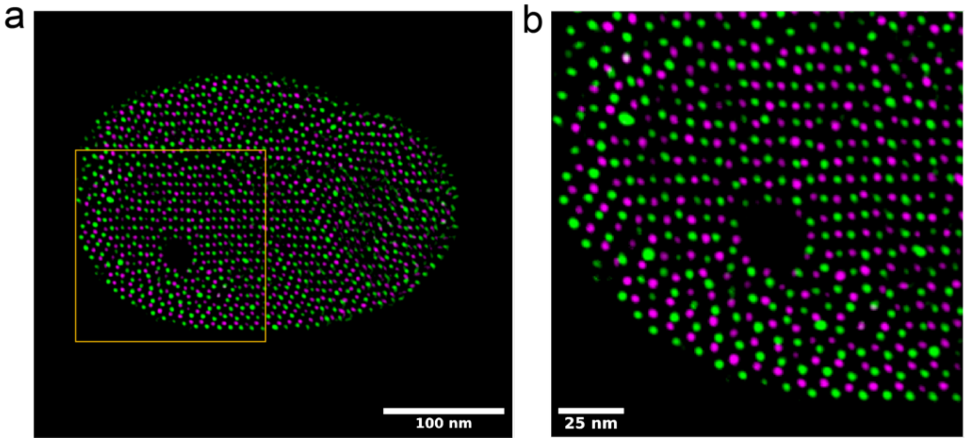


**Supplementary Fig. 18 | Reconstructed two consecutive plane layers near the top of the crystal.** (a) Two plane layers plotted in purple and green. (b) Zoomed-in image shows that near the edge of the crystal, a hollow site stacking characteristic in fcc/hcp structures is observed.

**11. PGNP clusters assembled from 25nm Au PGNPS**

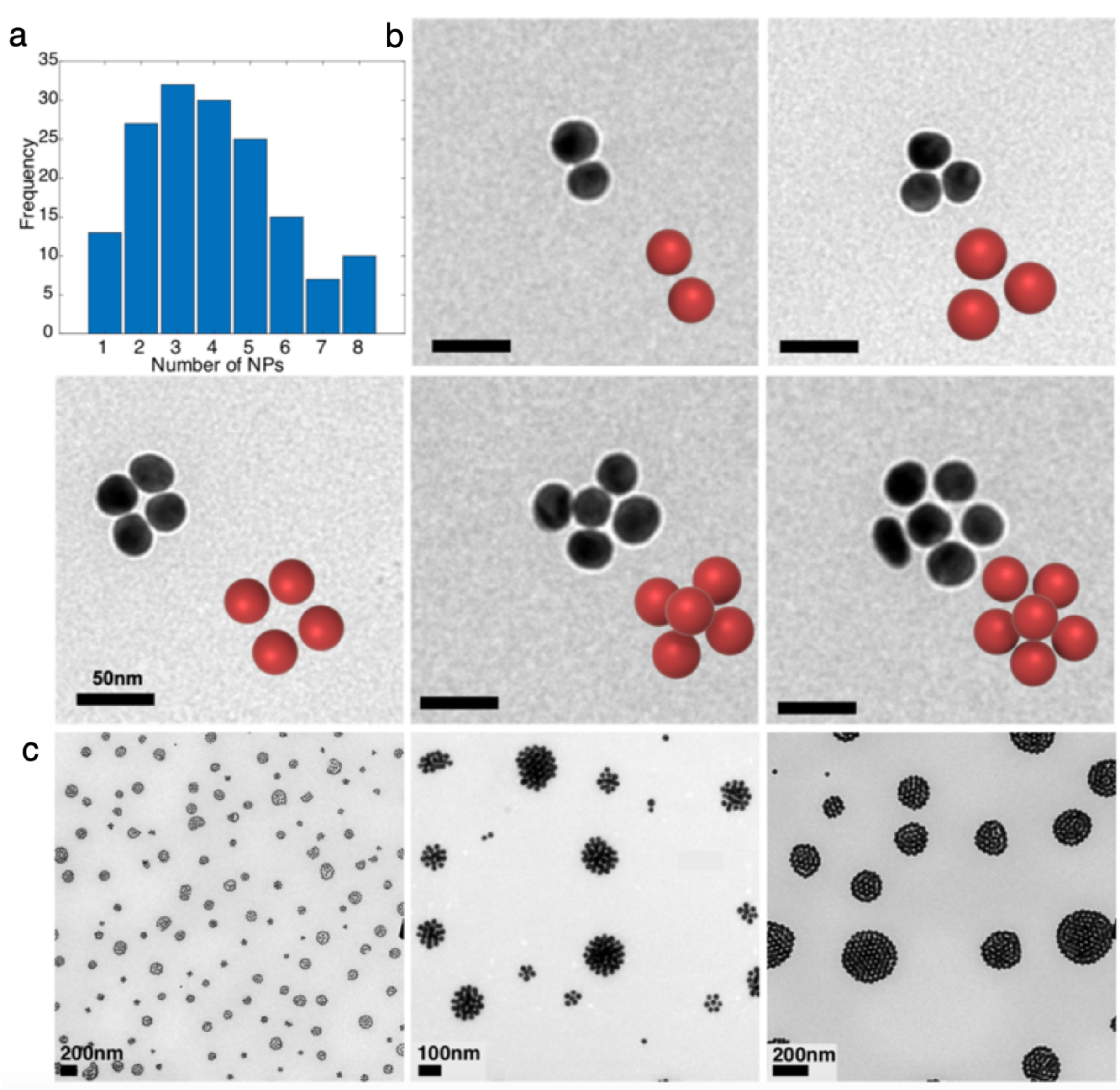


**Supplementary Fig. 19 | Assemblies of 25nm (6k) Au NPs.** (a) Histogram of NP clusters. (b) TEM images of Au NP clusters with various sizes. (c) TEM images of assembled Au NPs with different configurations.

## 12. PGNP clusters assembled on patterned substrates

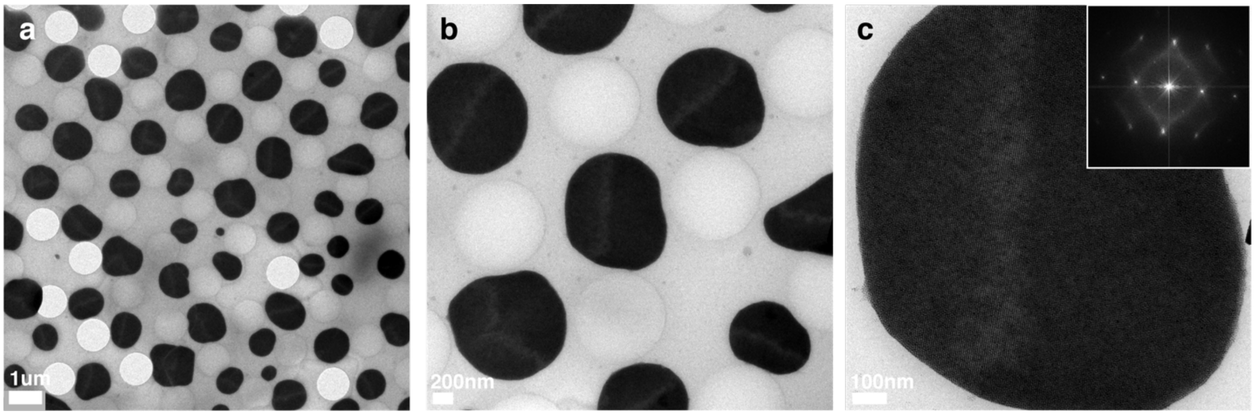


**Supplementary Fig. 20 | PNGP crystals formed on templated carbon films.** (a) A large-area of orthogonally arranged crystals assembled from Au-$PS_{0.60}$ NPs. (b-c) Zoomed-in TEM and FFT images show a bcc structure.

**Supplementary References:**


1. Peng S, Lee Y, Wang C, Yin H, Dai S, Sun S. A facile synthesis of monodisperse Au nanoparticles and their catalysis of CO oxidation. Nano Research. 2008;1(3):229-34.
2. Ye X, Zhu C, Ercius P, Raja SN, He B, Jones MR, et al. Structural diversity in binary superlattices self-assembled from polymer-grafted nanocrystals. Nature Communications. 2015;6:10052.
3. Korgel BA, Fitzmaurice D. Small-angle x-ray-scattering study of silver-nanocrystal disorder-order phase transitions. Physical Review B. 1999;59(22):14191.
4. Goodfellow BW, Patel RN, Panthani MG, Smilgies D-M, Korgel BA. Melting and sintering of a body-centered cubic superlattice of PbSe nanocrystals followed by small angle X-ray scattering. The Journal of Physical Chemistry C. 2011;115(14):6397-404.
5. Choi JJ, Bealing CR, Bian K, Hughes KJ, Zhang W, Smilgies D-M, et al. Controlling nanocrystal superlattice symmetry and shape-anisotropic interactions through variable ligand surface coverage. Journal of the American Chemical Society. 2011;133(9):3131-8.
6. Wang Z, Schliehe C, Bian K, Dale D, Bassett WA, Hanrath T, et al. Correlating superlattice polymorphs to internanoparticle distance, packing density, and surface lattice in assemblies of PbS nanoparticles. Nano letters. 2013;13(3):1303-11.
7. Goodfellow BW, Yu Y, Bosoy CA, Smilgies DM, Korgel BA. The Role of Ligand Packing Frustration in Body-Centered Cubic (bcc) Superlattices of Colloidal Nanocrystals. J Phys Chem Lett. 2015;6(13):2406-12.
8. Yun H, Yu J, Lee YJ, Kim J-S, Park CH, Nam C, et al. Symmetry Transitions of Polymer-Grafted Nanoparticles: Grafting Density Effect. Chemistry of Materials. 2019; 31(14): 5264-5273.
